# Antibacterial Activity of LCB10-0200 against *Klebsiella pneumoniae*

**DOI:** 10.3390/antibiotics10101185

**Published:** 2021-09-29

**Authors:** Sang-Hun Oh, Young-Rok Kim, Hee-Soo Park, Kyu-Man Oh, Young-Lag Cho, Jin-Hwan Kwak

**Affiliations:** 1 School of Life Science, Handong Global University, Pohang 37554, Korea; shoh@handong.edu (S.-H.O.); fred87@nate.com (Y.-R.K.); 2 School of Food Science and Biotechnology, Kyungpook National University, Daegu 41566, Korea; phsoo97@knu.ac.kr; 3 LegoChem Bioscience Inc., Daejeon 34302, Korea; kyuman@legochembio.com (K.-M.O.); young@legochembio.com (Y.-L.C.)

**Keywords:** LCB10-0200, *Klebsiella pneumoniae*, siderophore, cephalosporin

## Abstract

*Klebsiella pneumoniae* is one of the important clinical organisms that causes various infectious diseases, including urinary tract infections, necrotizing pneumonia, and surgical wound infections. The increase in the incidence of multidrug-resistance *K. pneumoniae* is a major problem in public healthcare. Therefore, a novel antibacterial agent is needed to treat this pathogen. Here, we studied the in vitro and in vivo activities of a novel antibiotic LCB10-0200, a siderophore-conjugated cephalosporin, against clinical isolates of *K. pneumoniae*. In vitro susceptibility study found that LCB10-0200 showed potent antibacterial activity against *K. pneumoniae*, including the beta-lactamase producing strains. The in vivo efficacy of LCB10-0200 was examined in three different mouse infection models, including systemic, thigh, and urinary tract infections. LCB10-0200 showed more potent in vivo activity than ceftazidime in the three in vivo models against the drug-susceptible and drug-resistant *K. pneumoniae* strains. Taken together, these results show that LCB10-0200 is a potential antibacterial agent to treat infection caused by *K. pneumoniae*.

## 1. Introduction

*Klebsiella pneumoniae*, an opportunistic pathogenic bacterium belonging to the Enterobacteriaceae group, can cause various infectious diseases in immunocompromised individuals [1,2]. Immunocompromised patients are at a high risk of contracting these infectious diseases, which are abundant in hospitals where immunocompromised individuals seek treatment [3,4,5]. Several antibiotics, including cephalosporin and carbapenem, have been used to treat infectious diseases caused by *K. pneumoniae*. However, most antimicrobial agents are currently ineffective in treating infections caused by these bacterial species because they are considered multidrug-resistant (MDR) pathogens and are on their way to becoming pan-drug resistant strains, which means that they show resistance to more than seven antibiotics simultaneously [6,7]. Therefore, it is necessary to develop a novel antibiotic to treat infectious diseases caused by antibiotic-resistant *K. pneumoniae*.

Beta-lactam antibiotics have been used to treat infection caused by *K. pneumoniae*, but some strains produce beta-lactamase, which renders these antibiotics ineffective [8,9,10]. In particular, the carbapenemase produced by *K. pneumoniae*, mainly KPC-2 and KPC-3, is a significant concern worldwide due to its rapid diffusion [11]. In particular, extended-spectrum beta-lactamase (ESBL) and metallo-beta-lactamase (MBL) producing *K. pneumoniae* can inactivate major beta-lactam antibiotics [12,13]. Most beta-lactamase genes are encoded in plasmids, which can be easily transferred to the same strain as well as to other strains.

Siderophore is an ion-chelating compound that transports iron into bacterium [14,15]. The Fe(III)-siderophore complexes bind receptor proteins, and these complexes are transported into cytoplasm [15]. Using this system, the siderophore—mediated drug delivery system was recently developed [16]. The siderophore-antibiotic conjugates are novel promising compounds able to treat infectious diseases caused by Gram—negative bacteria [16]. Cefiderocol and MB-1, siderophore—conjugated antibiotics developed by Shionogi and Pfizer, respectively, are conjugated with iron-adhering compounds that are structurally similar to the siderophore secreted by bacteria, which facilitates their recognition by bacterial surface receptors [17,18]. Siderophore-conjugated antibiotics can increase their membrane permeability, which in turn increases their antimicrobial activity [18,19].

**Figure 1:**
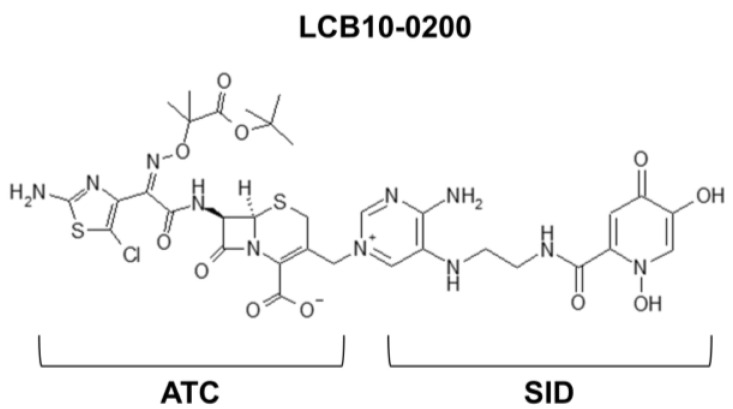
Structure of LCB10-0200. SID: siderophore, ATC: non-siderophore-conjugated cephalosporin.

LCB10-0200 (Figure 1), developed by LegoChem Biosciences (Daejeon, Korea), is a compound that conjugates the structure of the existing cephalosporin antibiotic (beta-lactam ring) and the siderophore structure of the microorganism and can easily enter into the microorganism using the siderophore receptor present on the cell wall surface of the microorganism [20]. Our previous study demonstrated that LCB10-0200 has potential activity against *Pseudomonas aeruginosa* [20]. In addition, in vitro susceptibility testing demonstrated that LCB10-0200 shows potent activity against various Gram—negative bacteria [20,21]. In the present study, we investigated the in vitro and in vivo activities of LCB10-0200 against cephalosporin-susceptible or cephalosporin-resistant *K. pneumoniae*.

## 2. Results

### 2.1. In Vitro Activity of LCB10-0200 against K. pneumoniae

Previous results demonstrated that LCB10-0200 was more active than ceftazidime, cefepime, aztreonam, and meropenem against clinically isolated *K. pneumoniae* [20]. We studied the in vitro activities of LCB01-0200, a siderophore compound alone (SID, C_12_H_14_N_6_O_4_), and a non-siderophore-conjugated compound (ATC). The SID had no antibacterial activity against the tested strains at the tested concentration. As shown in Table 1, LCB10-0200 was more active than SID and ATC against three *K. pneumoniae* strains.

We then examined the in vitro antibacterial activity of LCB10-0200 against beta-lactamase-producing pathogens to discover if there is an additional mechanism that is characteristic of beta-lactam antibiotics (siderophore mechanism). As shown in Table 2, LCB10-0200 (MIC range, 1–>32 mg/L) was more active than ceftazidime and ciprofloxacin against *K. pneumoniae*. Overall, these results demonstrated that LCB10-0200 has potent activities against *K. pneumoniae*.

### 2.2. In Vivo Activity of LCB10-0200 against K. pneumoniae

The in vivo activity of LCB10-0200 against *K. pneumoniae* was compared with that of ceftazidime using a mouse systemic infection model (Table 3). In this experiment, ceftazidime—susceptible and -resistant *K. pneumoniae* were used. In ceftazidime—susceptible *K. pneumoniae*, LCB10-0200 showed similar activity to ceftazidime. In ceftazidime-resistant *K. pneumoniae* ATCC700603 and 3835, LCB10-0200 (LD_50_ < 2.5 and 25.01, respectively) showed better efficacy than ceftazidime (LD_50_ > 20 and > 40, respectively) in the systemic mouse model.

**Figure 2:**
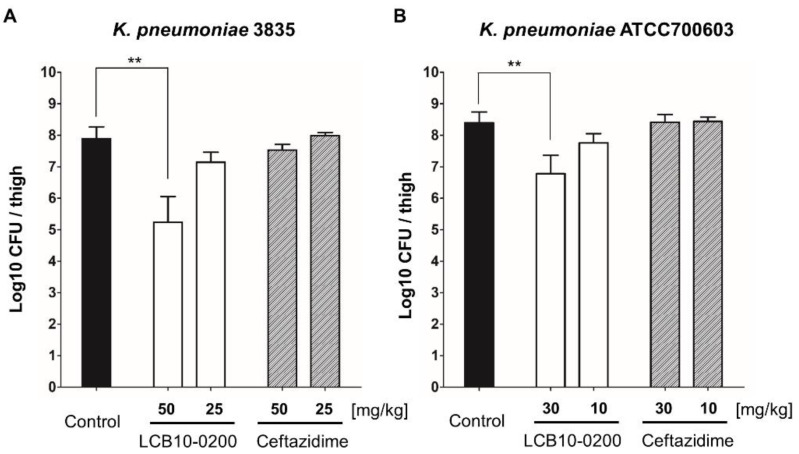
In vivo activity of LCB10-0200 in the thigh infection mouse model. Therapeutic efficacies of LCB10-0200 and ceftazidime in the thigh infection mouse model against two ceftazidime—resistant *K. pneumoniae* strains ((**A**) *K. pneumoniae* 3835; (**B**) *K. pneumoniae* ATCC700603). Each bar represents mean ± SD (standard deviation of the mean; *n* = 3; ** *p* < 0.01).

**Figure 3:**
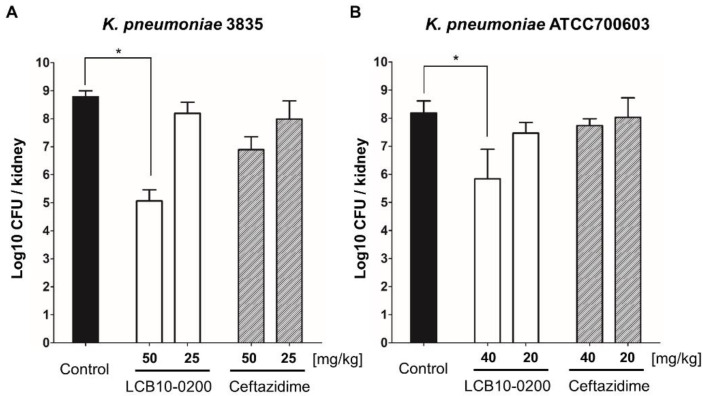
In vivo activity of LCB10-0200 in the urinary tract infection mouse model. Therapeutic efficacies of LCB10-0200 and ceftazidime in the urinary tract infection mouse model against two ceftazidime-resistant *K. pneumoniae* strains ((**A**) *K. pneumoniae* 3835; (**B**) *K. pneumoniae* ATCC700603). Each bar represents mean ± SD (standard deviation of the mean; *n* = 3; * *p* < 0.05).

In the thigh infection mouse model, LCB10-0200 also had a better activity compared to ceftazidime against both *K. pneumoniae* 3835 and *K. pneumoniae* ATCC700603 (Figure 2). In the urine infection mouse model, LCB10-0200 showed potent antibacterial activity against ceftazidime—resistant *K. pneumoniae* strains (Figure 3). Overall, these data strongly support that LCB10-0200 is a potent compound for the treatment of *K. pneumoniae* infection.

## 3. Discussion

In recent decades, bacteria have become resistant to most of the antibiotics used in hospitals [22]. In Korea, even colistin-resistant *Enterococcus* spp. have been reported [23]. Currently, plasmids carrying genes that are resistant to multiple antibiotics move easily between bacteria, and healthcare workers globally are experiencing significant difficulties in the treatment of these antibiotic-resistant bacteria [23,24].

The study of antibiotic-resistant Gram—negative bacterial strains has increased considerably in the past 10 years, and many research groups are working to develop novel antibiotics against MDR Gram—negative bacteria [25]. However, *P. aeruginosa*, *K. pneumoniae*, and *A. baumannii* inhibit the entry of antibiotics into bacterial strains while simultaneously obtaining various resistance mechanisms, including beta-lactamase secretion and efflux pump overexpression. These MDR bacteria are developing into pan-drug-resistant strains, thus necessitating the development of new antibiotics [26]. Previously, antibiotics to treat Gram—negative infections showed poor bacterial penetration, and researchers tried various ways to transport antimicrobial agents to the inside of cells. Several groups have developed a compound that mimics the structure of the siderophore used by bacteria to absorb iron or have conjugated antibiotics with a siderophore compound [18,19]. Since the structure of the synthesized compounds is similar to that of the siderophore, bacteria recognize it, allowing uptake. This “trojan horse” strategy has led to the development of antimicrobial agents that can be effective against MDR Gram—negative pathogens. MC-1 compounds developed by Pfizer and Cefiderocol manufactured by Shionogi are siderophore-conjugated antibiotic candidates that have shown excellent activity against multidrug-resistant Gram—negative bacteria, especially *P. aeruginosa* [18,19].

Our previous study and the present study demonstrated that LCB10-0200 also showed potent activity against *K. pneumoniae* when compared with the control drugs (Table 1) [20]. Moreover, LCB10-0200 was effective against the beta-lactamase-producing *K. pneumoniae* strains (Table 2). In addition, we tested whether LCB10-0200 could maintain its efficacy under various conditions, i.e., whether LCB10-0200 showed stable activity not only in normal cation-adjusted Mueller–Hinton broth but also in human-serum-supplemented medium and iron-depleted medium (data not shown). As the iron level increased in the broth media, the efficacy of LCB10-0200 decreased. This is because when the concentration of iron increases, the siderophore receptor is less expressed due to the increase of iron available for the bacteria. Because LCB10-0200 binding to iron depends on it entering the bacterium through its siderophore receptors, the probability that LCB10-0200 enters the bacterium is reduced.

In this study, we also tested the in vivo efficiency of LCB10-0200 in three mouse models. LCB10-0200 showed potent activity against *K. pneumoniae* strains in a systemic infection mouse model (Table 3). Importantly, the results obtained using the thigh and urinary tract infection mouse models showed that LCB10-0200 significantly decreased the bacterial counts in the thigh and kidneys, respectively (Figure 2 and Figure 3). In addition, the results of the previous hERG toxicity test confirmed that there was no toxicity at the highest concentration (300 μM) in the experiment [20]. Taken together, these results show the potential of LCB10-0200 as a therapeutic agent for beta-lactamase-producing *K. pneumoniae* strains.

While our previous and present studies provided in vivo and in vitro activities of LCB01-0200 against Gram—negative bacteria [27], we have not specified the molecular mechanism of LCB01-0200. Therefore, future studies will be carried out to better understand the mechanism of action of LCB01-0200 against Gram—negative bacteria, including *P. aeruginosa* and *K. pneumoniae*.

## 4. Materials and Methods

### 4.1. Antimicrobial Agents and Bacterial Strains

LCB10-0200 and ATC were synthesized by LegoChem Bioscience, Inc [20]. SID was obtained from WuXi AppTec Co. Ltd. (Tianjin, China). Ceftazidime and ciprofloxacin were purchased from Sigma-Aldrich (St. Louis, MO, USA).

*K. pneumoniae* isolates producing beta-lactamases were isolated from several hospitals in Seoul, Korea, during 2002–2016. For in vivo experiments, one ceftazidime-susceptible strain, *K. pneumoniae* ATCC13883, and two ceftazidime-resistant strains, *K. pneumoniae* 3835 and *K. pneumoniae* ATCC700603, were used.

### 4.2. Minimum Inhibition Concentration (MIC) Determination

The MICs of LCB10-0200 were determined using a two-fold agar dilution method, as described by the Clinical and Laboratory Standards Institute [28,29]. Briefly, *K. pneumoniae* was grown on Mueller—Hinton agar (MHA, Difco, Sparks, MD, USA) plates, sub-cultured into cation-adjusted Mueller—Hinton broth (CAMHB, Difco, Sparks, MD, USA), and incubated for 18 h at 37 °C. After cultivation, the cultured bacteria were diluted to obtain a bacterial cell density of approximately 10^6^ CFU/mL. All test organisms were seeded in MHA plates containing serial dilutions of LCB10-0200 or chemicals, using a multi-pin inoculator to achieve an antimicrobial concentration of 10^4^ CFU/spot. The plates were incubated at 35 °C for 18–20 h and were examined for bacterial growth. MIC was defined as the lowest concentration of the antimicrobial agent that completely inhibited bacterial growth on agar plates, disregarding a single colony or a faint haze caused by the inoculum.

### 4.3. Systemic Infection Mouse Model

For the systemic infection model, ICR male mice (four-week-old male weighing 18 to 20 g) (Daehan Bio Link Co., Ltd., Eum sung Gun, Korea) were used. Mice were maintained in animal chambers kept at 23 ± 2 °C with 55% ± 20% relative humidity. A systemic infection mouse model was established as described previously [20]. *K. pneumoniae* strains were cultured in CAMHB for 18 h at 37 °C. For inoculation, the cultured bacteria were suspended in 0.9% NaCl containing 5% gastric mucin (Sigma-Aldrich). Groups of five male ICR mice were intraperitoneally (*i.p.*) injected with a single 0.5 mL dose of the bacterial suspension. The challenge inoculum was sufficient to kill 100% untreated control mice within 24 h after infection. LCB10-0200 and ceftazidime were subcutaneously administered at 1 and 4 h after the bacterial infection. Mortality was recorded for 7 days, and the median effective dose required to protect 50% of mice (ED_50_) was calculated using the probit method with BioStat version 6 (Walnut, CA, USA).

### 4.4. Thigh Infection Mouse Model

The thigh infection mouse model was conducted as described previously [20]. Six-week-old female ICR mice (23–26 g) were *i.p.* injected twice with cyclophosphamide (Sigma-Aldrich), i.e., on day 4 (150 mg/kg) and day 1 (100 mg/kg), before bacterial inoculation. *K. pneumoniae* were cultured on MHA plates for approximately 18 h at 35 °C, and bacterial colonies were suspended in 0.9% saline solution. Bacterial suspension (1 × 10^6^ CFU/mL) was intramuscularly injected (0.1 mL) into each thigh of the anesthetized mice. LCB10-0200 and ceftazidime (0.2 mL) were subcutaneously administered at 3 and 6 h after the bacterial challenge. The mice were sacrificed after 24 h. The thighs of each mouse were dissected, homogenized, serially diluted with saline, and plated onto MHA plates to count the number of residual bacteria. These data were analyzed using GraphPad Prism 5 (San Diego, CA, USA). Statistical differences were analyzed by the Student’s unpaired *t*-test. This experiment was performed in duplicate.

### 4.5. Urinary Tract Infection Mouse Model

The urinary tract mouse model was established as described previously, with a minor modification [20]. Five-week-old female ICR mice were transurethrally injected with 50 μL suspension (about 2 × 10^8^ CFU/mL) of *K. pneumoniae* ATCC700603 or *K. pneumoniae* 3835. The urethral meatus of the infected mice was clamped for 2–4 h to prevent urine flow. LCB10-0200 and ceftazidime were administered at 3 and 6 h after infection. The mice were sacrificed at 24 h after infection, and the number of viable bacterial cells in the kidneys was determined. These data were analyzed using GraphPad Prism 5. Statistical differences were analyzed by the Student’s unpaired *t*-test. This experiment was performed in duplicate.

### 4.6. Animal Ethical Approval

All animal experiments were conducted in accordance with the ethical guidelines of the Ethics Review Committee for Animal Experimentation at Handong Global University (Korea) (Protocol #HGU-2010-04 and #HGU-20151022-003).

## 5. Conclusions

In conclusion, our in vivo results demonstrated that LCB10-0200 has potent antibacterial activities against *K. pneumoniae*. The results of this study and previous studies show that LCB10-0200 is a promising novel candidate for treating infections caused by Gram—negative bacteria, including *K. pneumoniae*, *P. aeruginosa*, and *A. baumannii*. Further studies on the mechanism of action and resistance mechanism will provide useful data for the development of LCB10-0200.

## Figures and Tables

**Table 1:** Comparative in vitro activities of LCB01-0200, SID, and ATC against *Klebsiella pneumoniae*.

Bacterial Strains	Compound MIC (mg/L)		
	LCB10-0200	SID	ATC
*K. pneumoniae* ATCC 13883	0.125	>64	16
*K. pneumoniae* ATCC700603	2	>64	64
*K. pneumoniae* 3835	1	>64	64

LCD10-0200: a novel siderophore cephalosporin conjugate, SID: siderophore, ATC: non-siderophore-conjugated cephalosporin.

**Table 2:** Comparative in vitro activities of LCB10-0200 against *Klebsiella pneumoniae* producing beta-lactamases.

Strain	Beta-Lactamase	MIC (mg/L)		
		LCB10-0200	Ceftazidime	Ciprofloxacin
*K. pneumoniae* 49	KPC-2	8	>32	>32
*K. pneumoniae* 50	KPC-2	8	>32	>32
*K. pneumoniae* 51	KPC-2	4	>32	>32
*K. pneumoniae* 52	GES-5	1	>32	2
*K. pneumoniae* 53	NDM-1	>32	>32	>32
*K. pneumoniae* 54	OXA-232	2	>32	>32
*K. pneumoniae* 55	OXA-232	32	>32	>32

**Table 3:** In vivo activities of LCB10-0200 against systemic infection model in mice.

Microorganism Inoculum (CFU/mouse) ^a^	Antimicrobial Agents ^b^	MIC (mg/L)	ED_50_ (mg/kg) (95% Confidence Limits)
*K. pneumoniae* ATCC13883	LCB10-0200	0.125	<0.8
(5 × 10^7^)	Ceftazidime	0.125	<0.8
*K. pneumoniae* ATCC700603	LCB10-0200	0.5	<2.5
(5 × 10^7^)	Ceftazidime	8	>20
*K. pneumoniae* 3835	LCB10-0200	1	25.01 (11.37–50.01)
(2 × 10^8^)	Ceftazidime	64	>40

^a^ Bacterial strains were suspended in 0.9% saline solution containing 5% mucin solution. ^b^ Antibiotics at various dose regimens were administered by subcutaneous injection, at 1 and 4 h post-infection.

## Data Availability

The data presented in this study are available on request from the corresponding author.
